# Dioscin ameliorates inflammatory bowel disease by up‐regulating miR‐125a‐5p to regulate macrophage polarization

**DOI:** 10.1002/jcla.24455

**Published:** 2022-05-07

**Authors:** Lingyan Shi, Peichen Zhang, Ruifang Jin, Xiaowei Chen, Lemei Dong, Weichang Chen

**Affiliations:** ^1^ Department of Gastroenterology The First Affiliated Hospital of Soochow University Suzhou China; ^2^ 89657 Department of Gastroenterology The First Affiliated Hospital of Wenzhou Medical University Wenzhou China; ^3^ 89657 Department of Gastrointestinal Surgery The First Affiliated Hospital of Wenzhou Medical University Wenzhou China

**Keywords:** dextran sulfate sodium, dioscin, inflammatory bowel disease, lipopolysaccharide, miR‐125a‐5p

## Abstract

**Purpose:**

Dioscin has been proven to have anti‐cancer, anti‐inflammatory, and anti‐infection roles. However, the role of Dioscin in inflammatory bowel disease (IBD) and its related mechanisms is unclear and needs further study.

**Methods:**

The colitis model in mice was established. After Dioscin (20, 40, or 80 mg/kg) treatment, the colon length was measured by a ruler. Histopathology, inflammatory cytokines, gut permeability, tight junction proteins, macrophage infiltration, macrophage polarization, and miR‐125a‐5p level were detected by hematoxylin–eosin staining, enzyme‐linked immunosorbent assay, quantitative real‐time polymerase chain reaction (qRT‐PCR), FITC‐dextran, Western blot, and flow cytometry. *In vitro* experiments, after RAW264.7 cells induced by lipopolysaccharide (LPS)/interleukin‐4 (IL‐4), were treated with Dioscin and miR‐125a‐5p inhibitor, miR‐125a‐5p level, cell vitality, inflammatory cytokines, and M1/M2 marker genes were measured by qRT‐PCR and MTT assay.

**Results:**

Dioscin (20, 40, or 80 mg/kg) relieved DSS‐triggered colitis and restrained the serum and colon of pro‐inflammatory cytokines expression. Meanwhile, different concentrations' Dioscin weakened M1 macrophage polarization but facilitated tight junction protein expressions, M2 macrophage polarization, and miR‐125a‐5p level in colitic mice. Moreover, miR‐125a‐5p inhibitor reversed the modulation of Dioscin on miR‐125a‐5p expression, cell vitality, and inflammatory cytokines in lipopolysaccharide (LPS)‐induced RAW264.7 cells. We further discovered that Dioscin restrained M1 marker gene (CD16) expression while intensifying M2 marker genes (CD206 and Arginase‐1) expressions *in vitro*, which was reversed by miR‐125a‐5p inhibitor.

**Conclusion:**

Dioscin modulated macrophage polarization by increasing miR‐125a‐5p, thereby improving the intestinal epithelial barrier function and reducing IBD.

## Graphical abstract

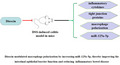

## 1 INTRODUCTION

Inflammatory bowel disease (IBD) is an immune‐mediated, chronic, and complex inflammatory disease of the digestive tract, including Chron's disease, ulcerative colitis, and uncertain colitis.^1^ The main clinical manifestations of IBD are diarrhea, abdominal pain, bloody stools, loss of appetite, weight loss, and lack of trace elements.^2^, ^3^ IBD patients' condition is repeated and protracted, which brings a heavy burden to patients. At present, the pathogenesis of IBD is believed to be caused by multiple factors, including environmental factors and specific genetic factors, in which the intestinal immune system responds too strongly to the normal intestinal flora or the intestinal flora disorder induces abnormal immune responses.^4^

Immune system disorder plays a pivotal role in the progression of IBD.^5^ As an important innate immune cell, macrophages can be divided into classically activated macrophages (M1 type) and alternatively activated macrophages (M2 type) according to their activation status, which generates a critical role in the development of IBD.^6^ In healthy intestines, the resident macrophages are M2‐type macrophages with an anti‐inflammatory effect, while the inflammatory M1‐type macrophages are dominant in the inflammatory intestinal mucosa of IBD.^7^, ^8^ More and more scientific studies have shown that the regulation of macrophage activation and polarization has a certain effect on the treatment of IBD.^9^, ^10^

In recent years, botanicals have played a crucial function in the treatment of various diseases due to their extensive pharmacological activities, sufficient drug sources, safety, stability, and lasting efficacy.^11^, ^12^ Dioscin is a steroidal saponin compound, which can be extracted from the rhizomes of certain Dioscoreaceae plants.^13^ Numerous studies have confirmed that Dioscin has the effects of protecting the liver,^14^ regulating the body's immune function,^15^ anti‐inflammatory,^16^ and anti‐tumor.^17^ Dioscin promotes anti‐tumor immunity via inhibiting macrophage M2 polarization,^15^ while reduces excessive inflammation via promoting macrophage M2 polarization.^18^ Also, Dioscin attenuates murine ulcerative colitis by regulating macrophage polarization.^19^ However, whether Dioscin can improve intestinal epithelial barrier dysfunction and inflammation by regulating macrophage polarization has not been reported yet.

Currently, various mouse models of IBD are indispensable tools for the preclinical validation of potential therapies.^20^, ^21^ Dextran sulfate sodium (DSS) method was used to induce severe colitis in mice after oral consumption of DSS, which was characterized by weight loss, diarrhea, bloody stool, ulcer formation, loss of epithelial cells, and infiltration of neutrophils.^22^ Therefore, this research assessed the role of Dioscin on macrophage polarization *in vitro* and *in vivo* and its molecular mechanism using the DSS‐induced colitis model in mice and RAW264.7 cells induced by lipopolysaccharide (LPS)/IL‐4.

## 2 METHODS

### 2.1 Ethics statement

The protocol on the animal was allowed in compliance with the regulations of the China Animal Care and Use Committee. This research was authorized by the Committee of Laboratory Animals of Nanfang Hospital (2019030020). We make every effort to mitigate the suffering of animals.

### 2.2 Dioscin

Dioscin was obtained from Sigma‐Aldrich (SMB00576, USA). We fully dissolved Dioscin in 0.5% sodium carboxymethyl cellulose (CMC‐Na, IS9000, Solarbio) solution in distilled water for *in vivo* experiments, and we fully dissolved Dioscin in 0.01% dimethyl sulfoxide (DMSO, D8371, Solarbio) for *in vitro* experiments.^23^

### 2.3 Preparation of DSS‐induced colitis model in mice and treatment

A total of 50 specific pathogen‐free (SPF) male C57BL/6 mice were provided from Beijing Vital River Laboratory Animal Technology Co., Ltd. (6–8 weeks old, weight 18–25 g), animal license number: SCXK (Beijing) 2019–0201. They were performed in the Nanfang Hospital SPF laboratory animal center. A 12‐h light/dark cycle was performed at a temperature of 21 ± 2°C and humidity of 30–70%. Mice were fed adaptively for 7 days.

The colitis model in mice was carried out according to the literature.^24^, ^25^ Fifty mice were randomly distributed to 5 groups, 10 in each group, namely the control group, DSS group, DSS + 20, DSS + 40, and DSS + 80. In the control group, mice drank blank distilled water from Day 1 to Day 10. In the DSS group, mice received 3% DSS (75027, Sigma‐Aldrich) to induce colitis in distilled water for 7 days and drank blank distilled water from Day 1 to Day 10. In the DSS + 20, DSS + 40, or DSS + 80 group, mice received 3% DSS in drinking distilled water from Day 1 to Day 7 and Dioscin was orally administered at doses of 20, 40, or 80 mg/kg daily from Day 1 to Day 10, respectively.^23^ On the 11th day, mice were euthanized by cervical dislocation. Next, the blood was obtained by enucleation of the eyeball. Then, mice were sacrificed by cervical dislocation, and colons of the mice were collected and measured using a ruler.

### 2.4 Histopathological assay

We used 10% formalin (G2162, Solarbio) to fix the colon tissues. The colon tissues were dehydrated with gradient ethanol and embedded in paraffin. The paraffin‐embedded tissues were sectioned, dewaxed, and hydrated. The slices were stained with hematoxylin solution (H8070, Solarbio). Then, we used 0.7% hydrochloric acid ethanol to differentiate for a few seconds and washed immediately. The slices were stained with 1% eosin solution (G1105, Solarbio) and then dehydrated with gradient ethanol. After the slices were transparent and sealed, we used an optical microscope (Z735515, Sigma) at magnification 100× to observe the slices. The pathological changes in the experimental colitis were evaluated using histopathological score.^26^ All the histological evaluations were performed and scored under blinded conditions.

### 2.5 Enzyme‐linked immunosorbent assay (ELISA)

Mouse IL‐1β kit (SEKM‐0002), IL‐6 kit (SEKM‐0007), and TNF‐α kit (SEKM‐0034) were derived from Solarbio. The blood was naturally coagulated and centrifuged at 4°C for 10 min to get serum samples. The reaction wells were reacted with 100 μl of standard and test samples and then placed at 37°C for 90 min. Next, 100 μl of the biotinylated antibody working solution was appended to the reaction wells and placed at 37°C for 60 min. Then, 100 μl of enzyme conjugate working solution was appended to the reaction wells and placed at 37°C for 30 min. The reaction wells were then reacted with 100 μl of the chromogenic substrate and developed color at 37°C away from light for 15 min. Finally, 50 μl of stop solution was appended to the reaction wells and the absorbance (450 nm) was determined using a microplate reader (SpectraMax iD5, Molecular Devices).

### 2.6 Quantitative real‐time polymerase chain reaction (qRT‐PCR)

Total RNAs from colon tissues and RAW264.7 cells were isolated using the TRIzol reagent (R1100, Solarbio). Subsequently, cDNAs were synthesized using the BeyoRT™ II cDNA first‐strand synthesis kit (D7168S, Beyotime) or miRNA cDNA first‐strand synthesis kit (KR211, TIANGEN). Then, we used the 2×Taqman PCR MasterMix (SR2110, Solarbio) and PCR instrument (CFX96, Bio‐Rad) to generate qRT‐PCR reactions. The results were normalized to GAPDH or U6, and the 2^−ΔΔC^ *^t^* method was utilized to count the mRNA expression levels.^27^ Primers are shown in Table 1.

**TABLE 1:** Gene sequence primers

Name	Forward primer(5′−3′)	Reverse primer(5′−3′)
TNF‐α	AGAGCTACAAGAGGATCACCAGCAG	TCAGATTTACGGGTCAACTTCACAT
IL‐1β	CAGGATGAGGACATGAGCACC	CTCTGCAGACTCAAACTCCAC
IL‐6	CTGCAAGAGACTTCCATCCAG	AGTGGTATAGACAGGTCTGTTGG
CD206	CTCTGTTCAGCTATTGGACGC	TGGCACTCCCAAACATAATTTGA
Arginase‐1	CTCCAAGCCAAAGTCCTTAGAG	GGAGCTGTCATTAGGGACATCA
CD16	TTTGGCCTACTACCCAAGGG	TTCGGTTCCTCTGTGGAATGA
miR‐125a‐5p	CCCTGAGACCCTTTAACCTGT	GCCCCTTCAAGCTCATTTCT
U6	GAAAGAAGACGCCGAGAAAGG	GGGAGATGTGGATCTATGTCGT
GAPDH	CGACTTCAACAGCAACTCCCACTCTTCTCC	TGGGTGGTCCAGGGTTTCTTACTCCTT

### 2.7 Intestinal epithelial permeability *in vivo*

The flux of FITC‐dextran (4kDA, 46994, Sigma‐Aldrich) is a classic biomarker for measuring intestinal epithelial permeability. After fasting for 12 h in each group, 20 mg/kg FITC‐dextran was administered to the mice and the mice were treated for 4 h before sacrifice. After that, blood samples were collected after sacrifice and coagulated at room temperature for 1 h. Finally, the concentration of FITC‐dextran was evaluated at the excitation wavelength (480 nm) and emission wavelength (520 nm) using the microplate reader.

### 2.8 Western blot

We used the RIPA buffer (abs9229, Absin) to lyse the colonic tissues. Next, the protein concentration was evaluated using the BCA kit (A045‐4‐2, Jiancheng, China). After that, the protein extract was electrophoresed and transferred to the nitrocellulose membrane (abs932, Absin). After blocking, the membranes were reacted with Occludin (1:1000, 59 kDa, ab216327, Abcam), ZO‐1 (1:3000, 187 kDa, ab96587, Abcam), Claudin1 (1:8000, 19 kDa, ab180158, Abcam), and GAPDH (1:10,000, 36 kDa, ab181602, Abcam) overnight at 4°C. They were then incubated with goat anti‐rabbit (1:5000, ab6721 Abcam, UK) at 37°C for 1 h. Finally, the blots were visualized using the ECL reagent (PE0010, Solarbio) under the gel imaging system (A44114, Invitrogen). The results were normalized to GAPDH.

### 2.9 Isolation of colonic lamina propria mononuclear cells (LPMCs)

First, the colon tissues were washed with prepared phosphate buffer (PBS, D1040, Solarbio) and cut into small pieces. Next, the colonic mass was incubated with PBS without Ca^2+^ and Mg^2+^ supplemented with 5 mM EDTA (E6758, Sigma‐Aldrich) and 1 mM dithiothreitol (43815, Sigma‐Aldrich) at room temperature with gentle shaking for 30 min. Subsequently, the above colon segments were incubated in RPMI 1640 medium (R8758, Sigma‐Aldrich) supplied with 1 mg/ml collagenase IV (C5138, Sigma‐Aldrich) at 37°C with gentle shaking for 1 h. After the supernatant was filtered and centrifuged, we used percoll gradient (40%–75%) to stratify the filtered cells and centrifuge them at 1000 g for 20 min. LPMCs were re‐suspended in PBS for flow cytometry assay.

### 2.10 Flow cytometry

The colonic LPMCs were reacted with anti‐mouse F4/80‐PE (ab237335, Abcam) and anti‐mouse CD11b‐APC (ab25482, Abcam) at 4°C for 40 min to analyze the infiltration of macrophages. Subsequently, the above‐mentioned cells were washed with PBS and then placed in a BD FACS Calibur flow cytometer (USA) for result analysis.

To perform macrophage polarization assay, macrophages were further gated to determine F4/80^+^CD11c^+^ M1‐like macrophages, and F4/80^+^CD206^+^ M2‐like macrophages in F4/80^+^CD11b^+^ (Figure 2G,H). Cell suspensions were stained with anti‐mouse F4/80‐PE (ab237335, Abcam), anti‐mouse CD206‐FITC (MA5‐16870, Invitrogen), and anti‐mouse CD11c‐APC (17‐0114‐82, Invitrogen). In the CD11b^+^F4/80^+^ cell population, cells that were F4/80^+^CD11c^+^ were defined as M1‐like macrophages, while those that were F4/80+CD206+ were defined as M2‐like macrophages, respectively.

### 2.11 Cell culture and grouping

Mouse mononuclear macrophage leukemia cells (RAW264.7) were obtained from WHELAB (China) and cultivated in RPMI1640 complete medium (M0201A, WHELAB) at 37°C with 5% CO_2_ incubator (3111, THERMO).

### 2.12 Cell transfection

MiR‐125a‐5p inhibitor (I, miR20000135‐1‐5) and inhibitor control (IC, miR2N0000002‐1‐5) were obtained from RiboBio (China). MiR‐125a‐5p I and IC were transfected into RAW264.7 cells, and the transfection efficiency was determined by qRT‐PCR after 48 h.

### 2.13 Cell grouping

To observe the effect of Dioscin and miR‐125a‐5p I on LPS‐induced RAW264.7 cells, the experiments were assigned into the Control group (untreated), LPS group (cells were exposed to 100 ng/ml LPS [L4391, Sigma‐Aldrich] for 24 h),^28^ LPS + Dioscin group (cells were pre‐treated with 150 ng/ml Dioscin for 24 h, and then exposed to 100 ng/ml LPS for 24 h),^23^ LPS + Dioscin + IC/LPS + Dioscin + I group (cells transfected with IC or I were pre‐treated with 150 ng/ml Dioscin for 24 h, and then subjected to 100 ng/ml LPS for 24 h), and LPS + I group (cells transfected with I were subjected to 100 ng/ml LPS for 24 h). Next, to investigate the effect of Dioscin and miR‐125a‐5p I on IL‐4‐induced RAW264.7 cells, the experiments were separated into the Control group, IL‐4 group (cells were incubated with 20 ng/ml recombinant mouse IL‐4 [ab259406, Abcam]),^29^ IL‐4 + Dioscin group (cells were exposed to 150 ng/ml Dioscin for 24 h, and then incubated with 20 ng/ml IL‐4 for 24 h), IL‐4 + Dioscin + IC/IL‐4 + Dioscin + I group (cells transfected with IC or I were exposed to 150 ng/ml Dioscin for 24 h and then incubated with 20 ng/ml IL‐4 for 24 h), and IL‐4 + I group (cells transfected with I were incubated with 20 ng/ml IL‐4 for 24 h).

### 2.14 Cell vitality assay

MTT kit (C0009S, Beyotime) was utilized to determine the RAW264.7 cell vitality. RAW264.7 cells (5 × 10^4^) were treated based on the above groups. Next, we used the 10 μl MTT solution to treat cells for 4 h in an incubator. After that, cells were reacted with 100 μl formazan solution for 3 h in an incubator. The absorbance (570 nm) was determined using the microplate reader.

### 2.15 Statistical analysis

Graphpad Prism 8.0 (GraphPad Software Inc.) was employed for statistical analysis. The measurement data were shown as mean ± standard deviation from at least three biological replicates with three technical replicates. The differences among multiple groups were determined via one‐way analysis of variance followed by Tukey's *post hoc* test. Statistical significances were considered to be *p* < 0.05.

## 3 RESULTS

### 3.1 Dioscin relieved DSS‐triggered colitis and restrained the pro‐inflammatory cytokines expression of serum and colon

**FIGURE 1:**
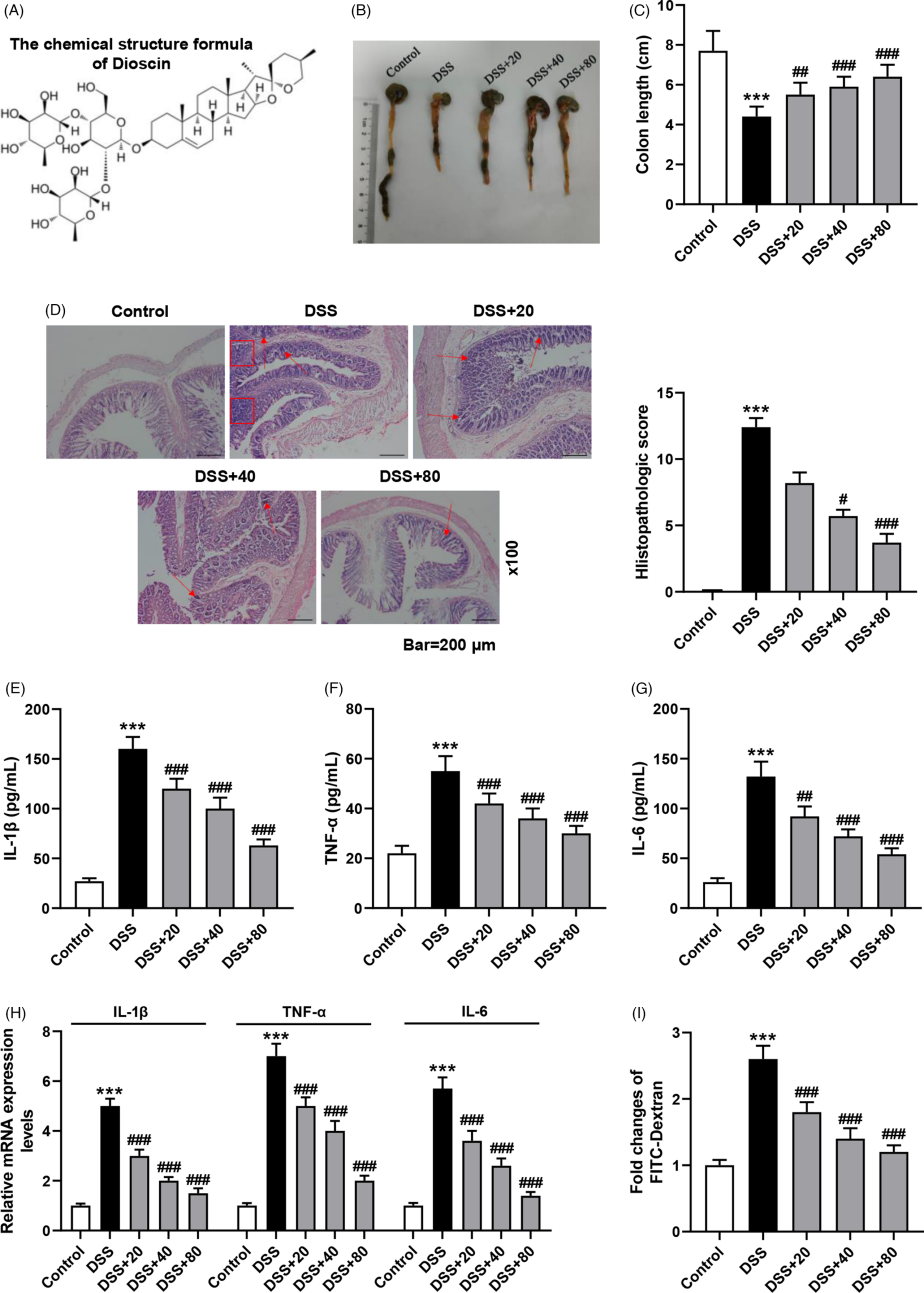
Dioscin relieved DSS‐triggered colitis and restrained the serum and colon of pro‐inflammatory cytokines expression in a dose‐dependent manner. (A) The chemical structure of Dioscin. (B,C) Impact of Dioscin on the colon length of colitic mice was measured by ruler (*n* = 10 per group). (D) Impact of Dioscin on histopathology of distal colon from colitic mice was detected by hematoxylin–eosin staining (magnification ×100, scale bar = 200 μm). Infiltration of inflammatory cells and tissue injury (arrow and quadrangle). (E–G) Impact of Dioscin on pro‐inflammatory cytokines expression in the serum of colitic mice was detected by enzyme‐linked immunosorbent assay. (H) Impact of Dioscin on pro‐inflammatory cytokines expression in the colon tissues of colitic mice was detected by quantitative real‐time polymerase chain reaction (qRT‐PCR). Expression levels were normalized with glyceraldehyde‐3‐phosphate dehydrogenase (GAPDH). (I) Impact of Dioscin on gut permeability in the serum of colitic mice was detected by FITC‐dextran. The dose of Dioscin: 20, 40, or 80 mg/kg. All experiments have been performed in triplicate and data were expressed as mean ± standard deviation (SD). ^***^ *p* < 0.001 vs. Control group; ^##^ *p* < 0.01, ^###^ *p* < 0.001 vs. dextran sulfate sodium (DSS) group. The figure represents at least 6 animal numbers/group

Figure 1A showed the chemical structure of Dioscin. Next, we measured the colon length and confirmed that the colon of the DSS group was largely shorter than the control group, while 20, 40, and 80 mg/kg Dioscin obviously increased colon length, especially in the high‐dose group of Dioscin (Figure 1B,C, *p* < 0.01). The H&E staining data manifested that there was massive infiltration of inflammatory cells and injury to the colonic tissues of the DSS group relative to the control group, while different concentrations of Dioscin largely alleviated the above symptoms and the high dose was the most significant (Figure 1D). Mice treated with Dioscin (20, 40, and 80 mg/kg) had significant lower histopathological scores than those treated with 3% DSS (Figure 1D, *p* < 0.05). The ELISA assay indicated that the levels of IL‐1β, TNF‐α, and IL‐6 in the serum of colitic mice were greatly decreased by different concentrations of Dioscin (Figure 1E–G, *p* < 0.01). Consistently, similar changes in pro‐inflammatory cytokines in the colonic tissues of colitic mice which were detected by qRT‐PCR got the same results (Figure 1H, *p* < 0.001). In addition, the data manifested that Dioscin weakened the flux of FITC‐dextran in the serum of colitic mice in a dose‐dependent manner (Figure 1I, *p* < 0.001).

### 3.2 Dioscin protected the tight junction proteins of colitic mice

**FIGURE 2:**
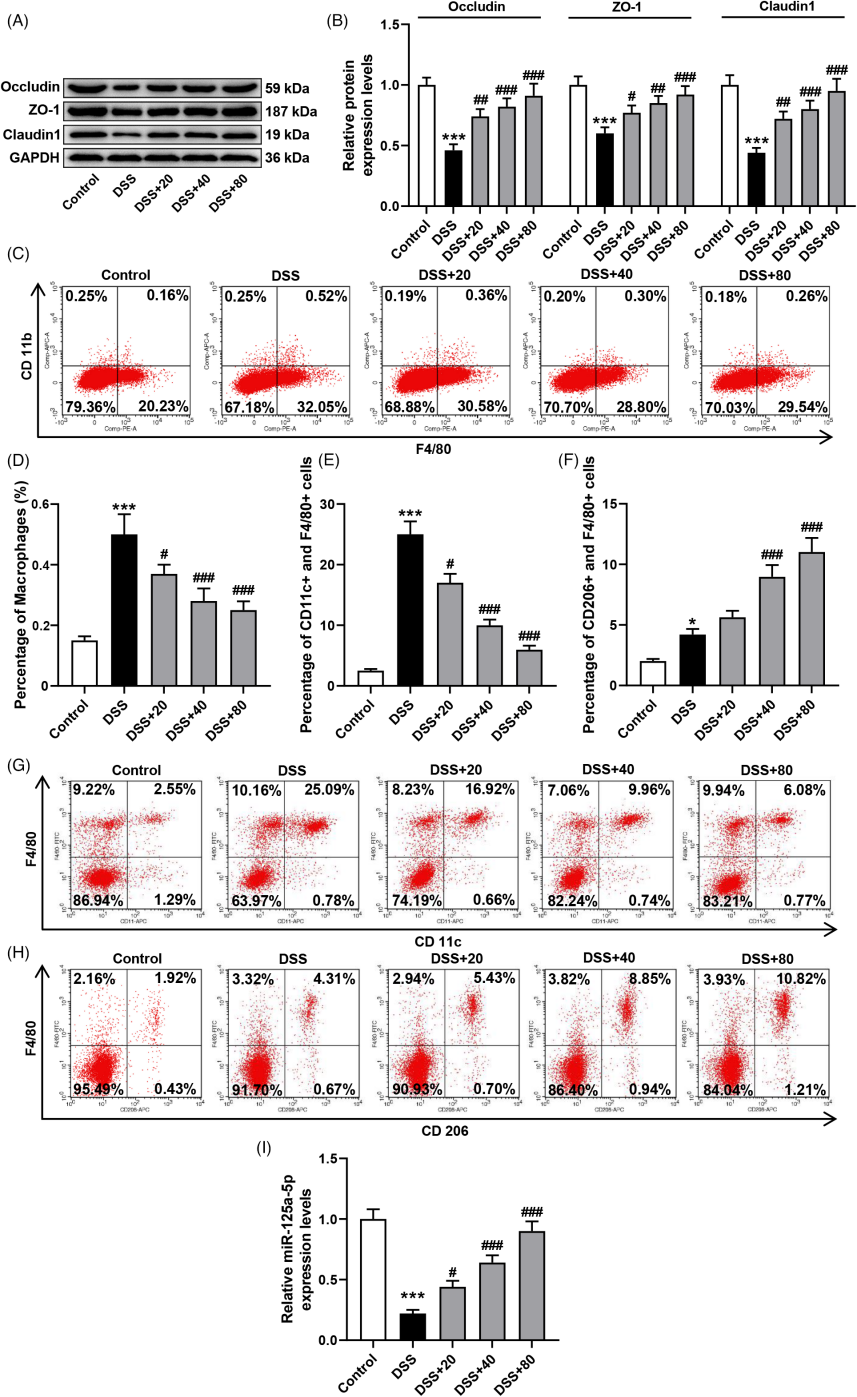
Different concentrations' Dioscin weakened M1 macrophage polarization but facilitated tight junction protein expressions and M2 macrophage polarization. (A,B) Impact of Dioscin on tight junction protein expressions in the colon tissues of colitic mice was detected by Western blot. Expression levels were normalized with GAPDH. (C,D) Impact of Dioscin on macrophage infiltration was detected by flow cytometry. (E‐H) Impact of Dioscin on macrophage polarization was detected by flow cytometry. (I) Impact of Dioscin on the miR‐125a‐5p level in the colon tissues of colitic mice was detected by qRT‐PCR assay. Expression levels were normalized with U6. The dose of Dioscin: 20, 40, or 80 mg/kg. All experiments have been performed in triplicate and data were expressed as mean ± standard deviation (SD). ^***^ *p* < 0.001 vs. Control group; ^#^ *p* < 0.05, ^##^ *p* < 0.01, ^###^ *p* < 0.001 vs. DSS group. The figure represents 6 animal numbers/group

In this research, we determined the tight junction protein expressions of colitic mice and manifested that the expressions of Occludin, ZO‐1, and Claudin1 were largely attenuated in the colonic tissues of the DSS group than the control group (Figure 2A,B, *p* < 0.001). On the contrary, Dioscin intensified the above protein expressions in the colonic tissues of DSS‐triggered colitic mice in a dose‐dependent manner (Figure 2A,B, *p* < 0.05).

### 3.3 Dioscin weakened macrophage infiltration and M1 macrophage polarization but facilitated M2 macrophage polarization in colitic mice

The flow cytometry assay manifested that the percentage of macrophages in the colon was largely elevated in the DSS group relative to the control group, while Dioscin obviously restrained the infiltration of macrophages (Figure 2C,D, *p* < 0.05). Then, we explored the function of Dioscin on the polarization of colonic macrophages and demonstrated that the percentage of M1 macrophages (F4/80+ CD11c+) and M2 macrophages (F4/80+ CD206+) in colonic macrophages was enhanced in the DSS group relative to the control group (Figure 2E–H, *p* < 0.05). Different concentrations' Dioscin obviously weakened the up‐regulation of the percentage of M1 macrophages (F4/80+ CD11c+) but further facilitated the up‐regulation of the percentage of M2 macrophages (F4/80+ CD206+) (Figure 2E–H, *p* < 0.05).

### 3.4 Dioscin intensified the miR‐125a‐5p expression in colitic mice

As displayed in Figure 2I, the miR‐125a‐5p level was largely restrained in the colonic tissues of the DSS group than the control group (*p* < 0.001). Interestingly, Dioscin greatly elevated the miR‐125a‐5p level of the colonic tissues in a dose‐dependent manner (Figure 2I, *p* < 0.05).

### 3.5 MiR‐125a‐5p I reversed the promotion of Dioscin on miR‐125a‐5p expression and cell vitality in LPS‐mediated RAW264.7 cells

**FIGURE 3:**
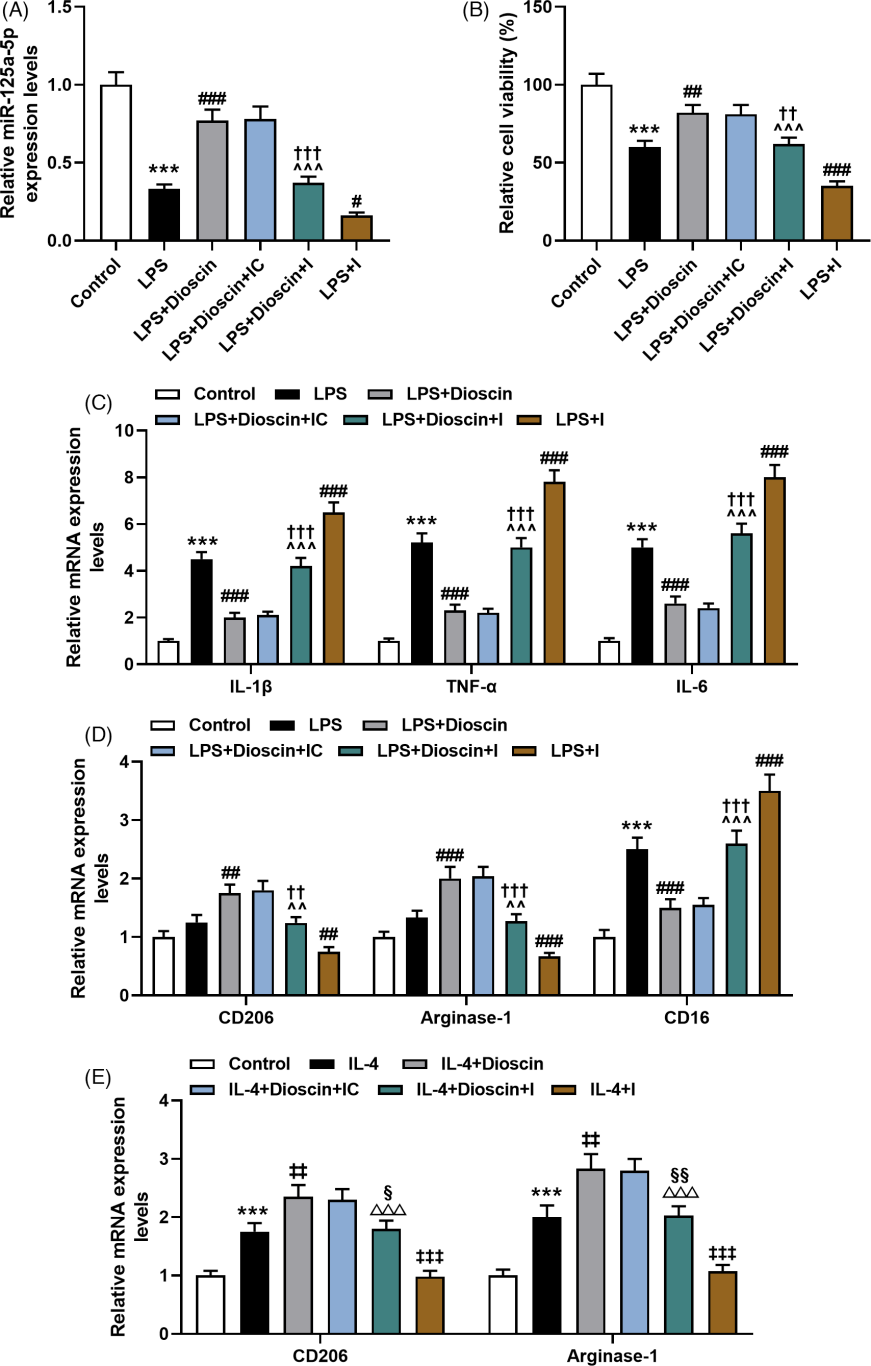
MiR‐125a‐5p inhibitor reversed the regulation of Dioscin on the miR‐125a‐5p level, cell vitality, inflammatory cytokines, and macrophage polarization *in vitro*. (A) Impacts of Dioscin and miR‐125a‐5p inhibitor (I) on the miR‐125a‐5p level in lipopolysaccharide (LPS)‐induced RAW264.7 cells were detected by qRT‐PCR assay. Expression levels were normalized with U6. (B) Impacts of Dioscin and miR‐125a‐5p inhibitor on the cell vitality in LPS‐induced RAW264.7 cells were detected by 3‐(4,5‐dimethylthiazol‐2‐yl)‐2,5‐diphenyltetrazolium bromide (MTT) assay. (C) Impacts of Dioscin and miR‐125a‐5p inhibitor on the inflammatory cytokines in LPS‐induced RAW264.7 cells were detected by qRT‐PCR assay. Expression levels were normalized with GAPDH. (D) Impacts of Dioscin and miR‐125a‐5p inhibitor on M1/M2 marker genes in LPS‐induced RAW264.7 cells were detected by qRT‐PCR assay. Expression levels were normalized with GAPDH. (E) Impacts of Dioscin and miR‐125a‐5p inhibitor on M2 marker genes in interleukin‐4 (IL‐4)‐induced RAW264.7 cells were detected by qRT‐PCR assay. Expression levels were normalized with GAPDH. Dioscin concentration: 150 ng/ml. All experiments have been performed in triplicate and data were expressed as mean ± standard deviation (SD). ^***^ *p* < 0.001 vs. Control group; ^##^ *p* < 0.01, ^###^ *p* < 0.001 vs. LPS group; ^^^^ *p* < 0.01, ^^^^^ *p* < 0.001 vs. LPS + I group; ^††^ *p* < 0.01, ^†††^ *p* < 0.001 vs. LPS + Dioscin + inhibitor control (IC) group; ^‡‡^ *p* < 0.01, ^‡‡‡^ *p* < 0.001 vs. IL‐4 group; ^ΔΔΔ^ *p* < 0.001 vs. IL‐4 + I group; ^§^ *p* < 0.05, ^§§^ *p* < 0.01 vs. IL‐4 + Dioscin + IC group. The figure represents three biological replicates with three technical replicates

As exhibited in Figure 3A, we provided evidence to show that Dioscin notably upraised the level of miR‐125a‐5p in LPS‐induced RAW264.7 cells, while miR‐125a‐5p I was the opposite (*p* < 0.05). However, co‐treatment of Dioscin and inhibitor reversed the regulation of Dioscin/inhibitor on miR‐125a‐5p expression (Figure 3A, *p* < 0.001). The MTT assay exhibited that Dioscin augmented the LPS‐induced RAW264.7 cell vitality, while miR‐125a‐5p inhibitor presented the opposite result (Figure 3B, *p* < 0.01). Nevertheless, the regulation of Dioscin/I on the cell vitality was reversed by co‐treatment of Dioscin and inhibitor (Figure 3B, *p* < 0.01).

### 3.6 MiR‐125a‐5p I partially offset the inhibition of Dioscin on inflammatory cytokines in LPS‐mediated RAW264.7 cells

The data in Figure 3C manifested that Dioscin largely restrained the levels of IL‐1β, TNF‐α, and IL‐6 in LPS‐triggered RAW264.7 cells, while miR‐125a‐5p I was the opposite (*p* < 0.001). However, co‐treatment of Dioscin and I could block the effect of Dioscin/I on inflammatory cytokine expressions (Figure 3C, *p* < 0.001).

### 3.7 Dioscin restrained M1 macrophage polarization while intensifying M2 macrophage polarization in RAW264.7 cells, which was reversed by miR‐125a‐5p I

The results of this part of the research supported that the expression of the M1 marker gene (CD16) was greatly enhanced (Figure 3D, *p* < 0.001), and the expression of M2 marker genes (CD206 and Arginase‐1) was slightly elevated in LPS‐induced RAW264.7 cells. Dioscin treatment greatly restrained the LPS‐mediated CD16 expression but facilitated the CD206 and Arginase‐1 expressions (Figure 3D, *p* < 0.01). However, miR‐125a‐5p I produced the opposite result to Doscin (Figure 3D, *p* < 0.01). Moreover, co‐treatment of Dioscin and I reversed the regulation of Dioscin/I on LPS‐induced M1 and M2 marker genes (Figure 3D, *p* < 0.01).

In addition, for M2 polarization, RAW264.7 cells were stimulated with 20 ng/ml recombinant mouse IL‐4 for 24 h and found that IL‐4 elevated the CD206 and Arginase‐1 expressions and Dioscin further elevated the IL‐4‐triggered the above gene expressions, while miR‐125a‐5p I was the opposite (Figure 3E, *p* < 0.01). However, these effects were all overturned by co‐treatment of Dioscin and inhibitor (Figure 3E, *p* < 0.05).

## 4 DISCUSSION

At present, the non‐biological treatment of IBD can relieve symptoms but does not affect the overall course of IBD.^30^ In addition, although the biological therapy of monoclonal antibodies has a rapid response, it has many disadvantages and high costs, causing serious social and economic burden.^31^ However, botanicals are more effective in the treatment of chronic inflammation due to their multi‐component, multi‐target, and multi‐pathway characteristics.^32^ Therefore, it is of practical significance to explore the role of Chinese herbal medicine in the treatment of IBD.

Many natural substances have been proven to have therapeutic effects on inflammatory diseases. For example, Bergenin restrained the activation of macrophages by modulating the PPARγ/SIRT1/NF‐κB‐p65 pathway, thereby ameliorating DSS‐triggered colitis in mice.^33^ Berberine weakened macrophages polarized toward M1 by modulating the AKT1/SOCS1/NF‐κB pathway, thus alleviating the incidence of DSS‐triggered colitic mice.^34^ In this study, we evaluated the effect of Dioscin on DSS‐triggered colitis in mice and its underlying mechanism for the first time. The evidence clarified that after Dioscin treatment, the colon length of colitic mice was largely enhanced and Dioscin greatly reduced the inflammatory damage of the colon.

Based on the above results, we further detected the expression of inflammatory factors in serum and tissues of mice with colitis. Our research results exhibited for the first time that IL‐1β, TNF‐α, and IL‐6 levels in serum of colitic mice were greatly decreased by Dioscin. Consistently, similar changes in inflammatory cytokines in the colonic tissues of colitic mice which were detected by qRT‐PCR got the same results. It was indicated that Dioscin could weaken DSS‐induced inflammation in colitic mice. The study has revealed that DSS has a direct toxic effect on colonic epithelial cells, which can cause epithelial cell damage and increase intestinal permeability.^35^, ^36^ Therefore, we measured the intestinal permeability and found that Dioscin weakened the flux of FITC‐dextran in the serum of colitic mice. Next, we tested tight junction‐related proteins and clarified that Dioscin intensified the Occludin, ZO‐1, and Claudin1 expressions in the colonic tissues of DSS‐triggered colitic mice. It was indicated that Dioscin has the function of protecting the intestinal epithelium of colitic mice.

The results have clarified that the development of IBD was closely related to macrophage dysfunction.^37^, ^38^ M1 macrophages mainly secrete inflammatory factors including TNF‐α, IL‐1β, and IL‐6, which can promote inflammation and enhance pathogen clearance by increasing phagocytosis, intracellular killing, and other paracrine cytokines.^39^ M2 macrophages mainly secrete pro‐inflammatory factors to resist the body's inflammatory reaction.^40^ Lupeol pre‐treated M1 macrophages largely repressed the expression of pro‐inflammatory cytokines IL‐6, IL‐12, TNF‐α, and IL‐1β and enhanced the expression of anti‐inflammatory cytokine IL‐10, which was associated with the decreased expression of M1 macrophage marker CD86 and the increased expression of M2 macrophage marker CD206.^8^ In this research, Dioscin obviously restrained the infiltration of macrophages. Then, we probed the role of Dioscin on the polarization of colonic macrophages and demonstrated that Dioscin obviously weakened the up‐regulation of the percentage of M1 macrophages (F4/80+ CD11c+) but further facilitated the up‐regulation of the percentage of M2 macrophages in the colon. Moreover, Dioscin greatly elevated the miR‐125a‐5p level of the colonic tissues in colitic mice.

Ge et al.'s research has revealed that miR‐125a‐5p attenuated the IBD progression via targeting ETS‐1.^41^ The forced expression of miR‐125a‐5p weakened the expression of TNF‐α, IL‐12, and iNOS triggered by LPS, while miR‐125a‐5p inhibitor repressed the expression of Arg1 triggered by IL‐4.^42^ In addition, only one study exhibited that Dioscin ameliorated the metabolic disorder of glucose and lipid in patients with type 2 diabetes by intensifying the inhibition of miR‐125a‐5p on STAT3.^43^ Our study complemented the deficiencies of Dioscin and miR‐125a‐5p in IBD research. Dioscin upraised the miR‐125a‐5p level and cell vitality and restrained the IL‐1β, TNF‐α, and IL‐6 levels in LPS‐induced RAW264.7 cells, while miR‐125a‐5p inhibitor was the opposite. Nevertheless, the above effects were reversed by co‐treatment of Dioscin and miR‐125a‐5p inhibitor. We further observed that Dioscin greatly restrained the LPS‐triggered CD16 expression but facilitated the CD206 and Arginase‐1 expressions, which was reversed by co‐treatment of Dioscin and miR‐125a‐5p inhibitor.

IL‐4 has been proven to be a classic Th2 cytokine that can trigger M2 macrophage polarization.^44^ We discovered that Dioscin further enhanced the expressions of CD206 and Arginase‐1 after IL‐4 exposure, while miR‐125a‐5p inhibitor was the opposite. More importantly, these effects were all overturned by co‐treatment of Dioscin and miR‐125a‐5p inhibitor. All in all, Dioscin has a protective effect on DSS‐triggered colitis and LPS/IL‐4‐triggered RAW264.7 cell injury, which is achieved by restraining the inflammatory response and colonic macrophage infiltration, especially by regulating the polarization of macrophages.

## AUTHOR CONTRIBUTIONS

Lingyan Shi and Peichen Zhang substantially contributed to conception and design and involved in drafting the article or critically revising it for important intellectual content. Ruifang Jin, Xiaowei Chen, Lemei Dong, and Weichang Chen contributed to data acquisition, data analysis, and interpretation. All the authors contributed to the final approval of the version to be published and agreement to be accountable for all aspects of the work in ensuring that questions related to the accuracy or integrity of the work are appropriately investigated and resolved.

## CONFLICT OF INTEREST

The authors declare that there is no conflict of interest.

## Data Availability

The analyzed data sets generated during the study are available from the corresponding author on reasonable request.
